# Plantar Pressure and Radiographic Deformity at Diagnosis of Active Charcot Neuro-Osteoarthropathy as Potential Prognostic Markers for Time to Remission

**DOI:** 10.3390/jcm15155828

**Published:** 2026-07-25

**Authors:** Victoria E. L. Milbourn, Kerensa M. Beekman, Max Nieuwdorp, Tessa E. Busch-Westbroek, Sicco A. Bus, Nina L. Petrova, Jaap J. Van Netten

**Affiliations:** 1 Department of Rehabilitation Medicine, Amsterdam University Medical Center, University of Amsterdam, Meibergdreef 9, 1105 AZ Amsterdam, The Netherlands; v.e.l.milbourn@amsterdamumc.nl (V.E.L.M.); s.a.bus@amsterdamumc.nl (S.A.B.); 2 Amsterdam Movement Sciences, Rehabilitation & Development, Amsterdam University Medical Center, University of Amsterdam, 1105 AZ Amsterdam, The Netherlands; k.m.beekman@amsterdamumc.nl; 3 Department of Radiology and Nuclear Medicine, Amsterdam Movement Sciences, Amsterdam University Medical Center, University of Amsterdam, 1105 AZ Amsterdam, The Netherlands; 4 Department of Internal and Vascular Medicine, Amsterdam University Medical Center, University of Amsterdam, 1105 AZ Amsterdam, The Netherlands; m.nieuwdorp@amsterdamumc.nl; 5 Diabeter (Center Amsterdam), 1066 EC Amsterdam, The Netherlands; 6 Mike Edmonds Foot Unit, King’s College Hospital National Health Service Foundation Trust, London SE5 9RS, UK; nina.petrova@nhs.net; 7 School of Cardiovascular and Metabolic Medicine and Sciences, Faculty of Life Sciences and Medicine, King’s College London, London SE1 7EH, UK

**Keywords:** Charcot neuro-osteoarthropathy, plantar pressure, remission, radiographic assessment, diabetic foot, prognostic markers, midfoot deformity

## Abstract

**Background**: Active Charcot neuro-osteoarthropathy (CNO) requires prolonged offloading, yet no reliable markers exist to predict time to remission at diagnosis. This study examined whether plantar pressure and radiographic deformity at diagnosis are associated with time to remission, and whether these measures provided independent and complementary prognostic value. **Methods:** This retrospective cohort study included 20 adults with active CNO. Barefoot peak plantar pressure and five radiographic angles from weight-bearing lateral radiographs were assessed at diagnosis. Associations with time to remission were examined using Spearman correlation and receiver operating characteristic (ROC) analysis. **Results:** Median time to remission was 132 days (interquartile range [IQR] 102 to 155). Medial midfoot plantar pressure showed a moderate non-significant association with time to remission (ρ 0.42, *p* = 0.064, area under the curve (AUC) 0.71). Of three radiographic angles with significant associations after false discovery rate correction, talar declination showed the strongest association with time to remission (ρ 0.76, *p* < 0.001) and demonstrated good discriminative performance for delayed remission (AUC: 0.89, 95% CI: 0.74 to 1.00). Meary’s angle performed comparably (AUC 0.86, 95% CI 0.69 to 1.00), consistent with high collinearity between measures (ρ = −0.94). Adding plantar pressure to talar declination did not improve discriminative performance (combined AUC 0.89, DeLong *p* = 1.00). **Conclusions:** In this exploratory cohort, talar declination and Meary’s angle demonstrated good discriminative performance for delayed remission. Plantar pressure showed a positive association with time to remission that did not reach statistical significance and did not improve discriminative performance when combined with radiographic assessment. Radiographic measurement at diagnosis, specifically talar declination or Meary’s angle, may support prognostic discussions with patients and inform decisions around offloading duration, pending prospective validation.

## 1. Introduction

Charcot neuro-osteoarthropathy (CNO) is a serious complication of diabetes, characterized by progressive bone and joint destruction of the foot and ankle, occurring in an estimated 0.04% to 0.79% of people with diabetes [1,2,3]. It is most commonly associated with peripheral neuropathy and leads to significant structural deformity, ulceration, and amputation, with reported lower extremity amputation rates of up to 29% and five-year mortality rates following CNO diagnosis exceeding 40% [4,5,6]. Delayed diagnosis is common and has been associated with worse structural outcomes and longer treatment duration [7,8,9]. Management of the active phase centers on offloading the affected limb to reduce mechanical stress and allow the inflammatory episode to resolve, and international guidelines recommend continued offloading until clinical and radiographic remission criteria are met [10,11]. The duration of offloading varies considerably, with reported treatment times ranging from three to twelve months [12]. No reliable markers have been identified to predict time to remission or guide clinical management during this period [10].

Given the biomechanical nature of CNO, structural deformity and plantar pressure are the logical domains in which to look for prognostic information. Structural deformity is a defining feature of CNO [13,14]. Progressive worsening of sagittal plane alignment has been demonstrated over one and two years in patients with active CNO compared to people with diabetes and peripheral neuropathy without CNO, with significant changes in Meary’s angle, cuboid height, and calcaneal pitch [15]. In patients with established CNO midfoot deformity, radiographic parameters have been associated with ulceration risk [16], though evidence remains limited to small observational cohorts. Whether the degree of structural deformity present at diagnosis is associated with the duration of the active episode has not been examined.

As midfoot collapse concentrates mechanical load on plantar bony prominences, structural deformity and elevated plantar pressure are biomechanically linked. Plantar pressure is significantly elevated in patients with active CNO compared to those with neuropathy alone [17]. In patients with established CNO midfoot deformity, barefoot midfoot pressures are markedly higher than in those with comparable diabetic foot risk without CNO, with median values of 756 versus 146 kPa [18], and elevated midfoot pressure has been associated with ulceration risk in chronic CNO deformity [14]. Given the rarity of CNO and the practical difficulty of obtaining pressure measurements within the timeframe of the active phase, the prognostic value of plantar pressure at diagnosis has not been previously studied. Neither plantar pressure nor radiographic deformity has been examined as a marker of time to remission, and whether they provide independent or complementary prognostic information is unknown.

Active CNO requires prolonged immobilization that significantly impacts mobility, work, and quality of life, yet clinicians currently have no reliable way to indicate likely treatment duration at the time of diagnosis [10,19,20]. Knowing the likely duration of cast treatment at the time of diagnosis, even as an approximation, may help patients prepare practically and psychologically for the episode ahead. We aimed to determine whether barefoot plantar pressures and radiographic measures of deformity at diagnosis of active CNO are associated with time to remission and discriminate between fast and delayed remission. We further assessed whether combining both plantar pressure and radiographic angles improved discriminative performance for delayed remission. Given the rarity of active CNO and the challenges of assembling sufficiently large cohorts, this study was exploratory and findings are hypothesis-generating.

## 2. Materials and Methods

### 2.1. Study Design and Setting

This retrospective cohort study was conducted at Amsterdam University Medical Centre (Amsterdam UMC), a tertiary referral center for diabetes-related foot complications, between January 2015 and 26 January 2024. Routinely collected clinical, plantar pressure, and radiographic data from patients with active CNO managed within a specialized multidisciplinary diabetic foot service were reused. Of the 20 participants included, 18 had previously been enrolled in an ongoing prospective CNO study at the same institution (EudraCT: 2016-003594-17). For these participants, an opt-out procedure was applied in which an information letter describing reuse of their data was sent and participants were given four weeks to raise an objection, after which non-objecting participants were included. The remaining two participants were newly recruited and provided written informed consent prior to inclusion, for use of their clinical data for study purposes. In both groups, participants were contacted only after their CNO episode had resolved. These procedures were conducted in accordance with Amsterdam UMC’s institutional requirements for the reuse of health care data for research purposes. The study was approved by the Medical Ethics Review Committee of Amsterdam UMC (reference number 2024.0542).

### 2.2. Participants

Patients were identified from two sources: electronic patient record (EPR) searches using diagnostic codes for CNO, and screening of the plantar pressure database of the laboratory for clinical gait assessment. Patients were eligible if they had a confirmed diagnosis of diabetes mellitus and active CNO. Patients were excluded if they had undergone a major lower limb amputation, had active ulceration of the index limb at CNO diagnosis, or had a history of rheumatoid disease. Plantar pressure and radiographic data were required to be performed within three months of diagnosis. Baseline clinical and laboratory variables were extracted from the electronic patient record within the same timeframe. This timeframe was defined pragmatically to reflect routine clinical practice, in which radiographs are obtained at or close to diagnosis as part of standard assessment, while plantar pressure measurements may follow within the subsequent weeks as part of gait laboratory review.

Eligibility was confirmed by review of medical records. Baseline demographic and clinical data were extracted from the EPR, plantar pressure data from the gait laboratory database, and radiographic images from the hospital radiology archive. Baseline variables included age at diagnosis, sex, diabetes type and duration, presence of peripheral neuropathy and peripheral artery disease, history of foot ulceration, and CNO classification by Eichenholtz stage [21,22] and Sanders and Frykberg’s anatomical classification [23].

Data for the present study was extracted from routine clinical records and was not derived from trial-specific case report forms of the prior CNO study.

### 2.3. Plantar Pressure Assessment

**Figure 1:**
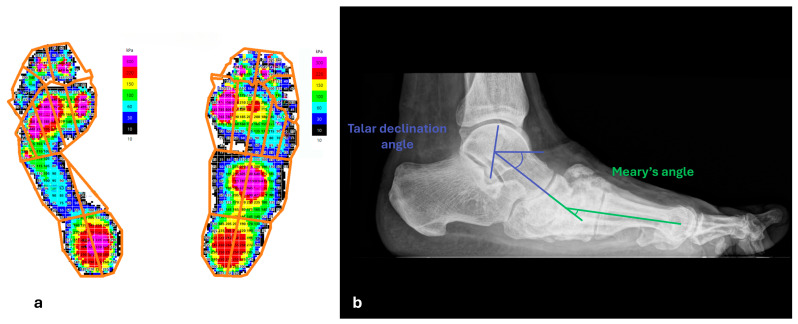
Plantar pressure regional masks of left foot and right foot (**a**) and representative radiographic measurements from a weight-bearing right foot radiograph (**b**) used in the present study, from a single participant at diagnosis. (**a**) Averaged maximum barefoot plantar pressure maps of both feet showing the 12 regional masks. The medial midfoot and lateral midfoot regions are indicated as the primary regions of interest on the right foot. (**b**) Weight-bearing lateral radiograph of the right foot with the measurements of the talar declination angle and Meary’s angle annotated. Additional radiographic measurements analyzed in the study (calcaneal pitch, cuboid height, and calcaneal-fifth metatarsal angle) are not shown for clarity.

Barefoot plantar pressure measurements were obtained at diagnosis and remission as part of routine clinical care using a Novel EMED-X pressure platform (Novel GmbH, Munich, Germany) measuring at a sampling frequency of 50 Hz. The gait laboratory follows a standardized two-step data collection protocol in which patients walked at a self-selected speed and a minimum of four valid trials per foot were recorded [24]. Peak plantar pressure (kPa) was calculated for each trial and averaged across valid trials for each region. The plantar surface was divided into 12 standardized anatomical regions using the manufacturer masking protocol (Figure 1a). Analyses were performed for three pre-specified regions: total foot, medial midfoot, and lateral midfoot. The midfoot regions were the primary areas of interest, as CNO most commonly affects the midfoot with collapse of the medial and lateral columns producing the characteristic structural deformity [23,25].

### 2.4. Radiographic Assessment

Plain foot radiographs were obtained at diagnosis and at remission as part of the standard clinical protocol of the multidisciplinary diabetic foot service. Each imaging sequence included anteroposterior, oblique, and weight-bearing lateral projections. Images were retrieved from the hospital radiology archive. Five parameters were assessed on weight-bearing lateral views: Meary’s angle, talar declination angle, calcaneal pitch, cuboid height, and the calcaneal–fifth metatarsal angle. Talar declination and Meary’s angle are illustrated in (Figure 1b). Meary’s angle was measured as the angle between a line bisecting the talar body, neck and head and the longitudinal axis of the first metatarsal, with negative values indicating plantar flexion of the forefoot relative to the talus. Talar declination was measured as the angle between the longitudinal axis of the talus and the horizontal weight-bearing surface. Calcaneal pitch was measured as the angle between the inferior border of the calcaneus and the horizontal weight-bearing surface. Cuboid height was measured as the perpendicular distance from the plantar aspect of the cuboid to a line connecting the plantar calcaneal tuberosity and the plantar aspect of the fifth metatarsal head, with negative values indicating plantar displacement below this line. The calcaneal–fifth metatarsal angle was measured as the angle between the longitudinal axis of the calcaneus and the longitudinal axis of the fifth metatarsal [26,27,28]. Measurements were performed using IDS7 (version 28.1; Sectra AB, Linköping, Sweden) with the musculoskeletal foot and ankle module.

All radiographic images were pseudonymized and presented in randomized order prior to measurement. Measurements were performed independently by a specialist podiatrist (VM) and a specialist musculoskeletal radiologist (KB). Inter-rater reliability was assessed in a randomly selected subsample of 12 participants, with one image per participant selected from either the diagnosis or remission timepoint using random number generation in Excel. KB was blinded to clinical outcomes and imaging timepoint. Where landmarks were obscured or not measurable, measurements were recorded as missing for that variable.

### 2.5. Outcome Definition (Remission)

The primary outcome was time to remission of active CNO, defined as the interval in days between the recorded date of CNO diagnosis and the date of confirmed remission. As part of the standard clinical pathway at Amsterdam UMC, remission was determined by multidisciplinary consensus within the specialist diabetic foot clinic, informed by clinical findings and radiographic assessment with input from the radiologist specializing in diabetic foot disease. The clinical decision was supported by documented resolution of local inflammatory signs including reduction in swelling and erythema, together with improvement in skin temperature asymmetry between the affected and contralateral foot. A skin temperature difference of 2 °C or less between limbs was used as a supportive indicator, consistent with published Charcot cohorts [29,30]. Radiographic consolidation, defined as the absence of new fractures, displacement or progressive structural collapse on serial plain radiographs, was considered alongside clinical findings. This approach is consistent with international guidance, which recommends combining clinical findings, skin temperature monitoring and imaging when determining remission, acknowledging that no single validated criterion exists [30]. The remission date was extracted retrospectively from the electronic patient record, defined as the date on which the multidisciplinary team documented that remission had been reached, and clinical management was progressed beyond immobilization.

### 2.6. Statistical Analysis

Statistical analyses were performed in R version 4.4.3 (R Foundation for Statistical Computing, Vienna, Austria). Continuous variables were summarized as mean (standard deviation) or median as appropriate. Categorical variables were summarized as counts and percentages.

Inter-rater reliability for radiographic measurements was assessed using intraclass correlation coefficients (ICC) based on a two-way random-effects model with absolute agreement, ICC(2,1). Values were interpreted as poor (less than 0.50), moderate (0.50 to 0.74), good (0.75 to 0.89), or excellent (0.90 or above) [31].

Associations between plantar pressure at diagnosis and time to remission, and between radiographic angles at diagnosis and time to remission, were assessed using Spearman rank correlation. Correlation magnitude was interpreted using pre-specified thresholds of very weak (less than 0.20), weak (0.20 to 0.39), moderate (0.40 to 0.59), strong (0.60 to 0.79), and very strong (0.80 or above). To account for multiple comparisons, false discovery rate (FDR) correction using the Benjamini–Hochberg method was applied separately to the plantar pressure correlation analyses (three comparisons) and the radiographic angle correlation analyses (five comparisons) [32].

Receiver operating characteristic (ROC) analysis was performed to evaluate the discriminative performance of plantar pressure variables and radiographic variables for delayed remission. No clinically validated threshold for delayed remission in active CNO currently exists. A median split of time to remission was therefore used to define delayed remission, providing two balanced comparison groups appropriate to the limited sample size. The resulting value (132 days) reflects this cohort’s distribution of remission times. Area under the ROC curve (AUC) with 95% confidence intervals, sensitivity, specificity, positive predictive value, negative predictive value, and optimal thresholds identified by the Youden index were calculated.

To examine whether combining plantar pressure and radiographic deformity at diagnosis improved discrimination for delayed remission, exploratory logistic regression models were constructed. Prior to modeling, collinearity between radiographic variables was assessed using Spearman correlation. Where collinearity was high, a single representative variable was selected based on the strength of its association with the outcome. Model performance was summarized using AUC with 95% confidence intervals and compared using DeLong’s test [33], performed using the pROC package in R [34].

A two-sided *p*-value of less than 0.05 was considered statistically significant. Given the small sample size inherent to research in this rare condition, all findings should be interpreted as hypothesis-generating rather than confirmatory.

As an exploratory sensitivity analysis, differences in time to remission across Sanders–Frykberg anatomical pattern groupings were assessed using the Mann–Whitney U test.

## 3. Results

### 3.1. Cohort Characteristics

Twenty-three participants were identified. Eighteen had previously enrolled in the prior CNO study, for whom the opt-out procedure was applied, and five were newly recruited participants approached for written informed consent. Of the five newly recruited participants, two did not return a signed consent form and one was subsequently excluded due to insufficient plantar pressure data, leaving 20 participants with active CNO included in the analysis (18 via opt-out, two via written informed consent). Baseline demographic and clinical characteristics are summarized in Table 1. The cohort was equally distributed by sex and CNO laterality. Mean age at diagnosis was 58 years and the majority had type 2 diabetes (85%). All participants had peripheral neuropathy and 60% had a history of foot ulceration on the index limb. Most cases were classified as Eichenholtz stage 1 (70%), with the remainder stage 0, defined as clinical signs of active CNO with normal plain X-rays but osseous abnormalities on advanced imaging [11]. At diagnosis, based on Sanders–Frykberg classification ten participants (50%) presented with pattern II tarsometatarsal involvement, seven (35%) with multiple pattern involvement, and the remaining three presented with patterns I, III and IV respectively. The pattern I and pattern IV cases represented the only participants with non-midfoot CNO.

### 3.2. Radiographic Measurement Reliability

In the subsample of 12 participants used to determine reliability of radiographic measurements, inter-rater reliability for radiographic measurements was good to excellent across all angles (ICC[2, 1] range 0.82 to 0.98). Reliability was highest for calcaneal pitch (ICC: 0.98, 95% CI 0.93 to 0.99) and cuboid height (ICC: 0.97, 95% CI 0.90 to 0.99). Reliability was lowest for talar declination (ICC: 0.82, 95% CI 0.51 to 0.94), with the lower confidence interval extended into the moderate range, reflecting greater variability in landmark identification for this measure. Meary’s angle (ICC 0.92, 95% CI 0.75 to 0.98) also demonstrated strong agreement. The calcaneal–fifth metatarsal angle showed equivalent reliability (ICC 0.92, 95% CI 0.73 to 0.98) based on assessment of 11 participants, as landmarks were not measurable in one case. Median absolute differences between raters ranged from 0.5 mm (cuboid height) to 3.5° (talar declination) across all five measures.

### 3.3. Time to Remission

Median time to remission was 132 days (IQR 102 to 155, range 89 to 285 days). Participants were classified as having delayed remission if time to remission exceeded 132 days.

### 3.4. Barefoot Plantar Pressure at Diagnosis and Remission

Barefoot plantar pressure measurements were obtained at a median 12 days after CNO diagnosis (IQR 2 to 21 days, range 0 to 59 days). Regional plantar pressure at diagnosis and remission, with baseline values stratified by remission group, is summarized in Table 2. Midfoot mean peak plantar pressure at diagnosis was higher in participants with delayed remission than in those with fast remission, most notably in the lateral midfoot (232 kPa versus 129 kPa). Plantar pressure in other regions showed little difference between groups.

Total peak plantar pressure at diagnosis was not associated with time to remission (Spearman ρ = 0.17, *p* = 0.465, FDR-adjusted *p* = 0.465). Medial midfoot peak plantar pressure showed a moderate positive correlation with time to remission (ρ = 0.42, *p* = 0.064, FDR-adjusted *p* = 0.158) and lateral midfoot peak plantar pressure showed a weak positive correlation (ρ = 0.37, *p* = 0.105, FDR-adjusted *p* = 0.158). The identical FDR-adjusted *p*-values for medial and lateral midfoot (both 0.158) reflect rounding following correction across three comparisons. Higher midfoot peak plantar pressure was generally associated with longer time to remission. Effect sizes were weak to moderate and variability between individuals was substantial. Spearman correlations for all predictors and time to remission are presented in Table 3.

Discriminative performance of plantar pressure variables for delayed remission is summarized in Table 4. Total peak plantar foot pressure showed high sensitivity at the Youden threshold (0.90) but poor specificity (0.50), indicating limited discriminative utility. Although lateral midfoot peak plantar pressure showed higher overall discrimination than medial midfoot peak plantar pressure (AUC 0.77 versus 0.71), the small sample size limits the number of achievable sensitivity/specificity combinations, and both regions reached their optimal Youden point at identical values (sensitivity 0.70, specificity 0.80) despite different thresholds. Confidence intervals were wide across all measures.

### 3.5. Radiographic Angles at Diagnosis and Remission

Radiographic angles at diagnosis and remission are summarized in Table 5. At diagnosis, the cohort demonstrated a pattern consistent with midfoot collapse, with a mean Meary’s angle of −9.7° (SD 11.4), mean talar declination of 25.4° (SD 7.6), and mean calcaneal pitch of 18.3° (SD 5.8). Sanders–Frykberg classification remained unchanged in 19 of 20 participants between diagnosis and remission.

Radiographic angles showed minimal change over the course of the episode. Mean changes between diagnosis and remission were small across all measures, ranging from −1.6° for Meary’s angle to 1.4° for talar declination, with individual variability exceeding the magnitude of the mean change in all cases.

Baseline radiographic deformity was strongly associated with time to remission for three of the five angles examined (Table 3). A more negative Meary’s angle was associated with prolonged time to remission (Spearman ρ = −0.69, *p* = 0.001, FDR-adjusted *p* = 0.002), as was higher talar declination (ρ = 0.76, *p* < 0.001, FDR-adjusted *p* < 0.001) and more negative calcaneal–fifth metatarsal angle (ρ = −0.65, *p* = 0.005, FDR-adjusted *p* = 0.008), indicating greater lateral column collapse. These associations remained statistically significant after FDR correction. Although the signs of the correlations differ between measures, this reflects differences in how each angle is defined rather than inconsistency in direction, as greater deformity in each case was associated with longer remission. Calcaneal pitch and cuboid height showed weak negative associations with time to remission (ρ = −0.39, *p* = 0.091 and ρ = −0.24, *p* = 0.312).

Discriminative performance of radiographic angles at diagnosis for delayed remission is summarized in Table 4. Talar declination showed the strongest discrimination (AUC 0.89, 95% CI 0.74 to 1.00), with sensitivity of 0.80 and specificity of 0.90 at a threshold of 25°. Meary’s angle showed comparable discrimination (AUC 0.86, 95% CI 0.69 to 1.00) with perfect sensitivity at the Youden threshold, though confidence intervals were wide across all measures, consistent with the small sample size. Calcaneal pitch and cuboid height showed poor to moderate discrimination, consistent with their weak Spearman coefficient correlations.

### 3.6. Relationship Between Plantar Pressure and Radiographic Deformity at Diagnosis

Only one association between peak plantar pressure and radiographic angles at diagnosis reached nominal significance. Medial midfoot peak plantar pressure demonstrated a moderate inverse association with Meary’s angle (ρ = −0.61, unadjusted *p* = 0.005). Higher medial midfoot peak plantar pressure was associated with greater arch collapse at diagnosis. This association did not remain significant after FDR correction (FDR-adjusted *p* = 0.075). No other associations between plantar pressure and radiographic angles reached significance before or after correction. Full correlation results are presented in Table A1.

### 3.7. Combined Prediction of Delayed Remission

Prior to modeling, Meary’s angle and talar declination were found to be highly collinear (Spearman ρ = −0.94), consistent with both measures reflecting the same underlying deformity. The difference in discriminative performance between talar declination and Meary’s angle was not statistically significant (DeLong *p* = 0.621). Talar declination was selected as the single radiographic predictor given its stronger association with time to remission. Medial midfoot peak plantar pressure was selected as the plantar pressure predictor based on its marginally stronger association with time to remission in the correlation analyses.

**Figure 2:**
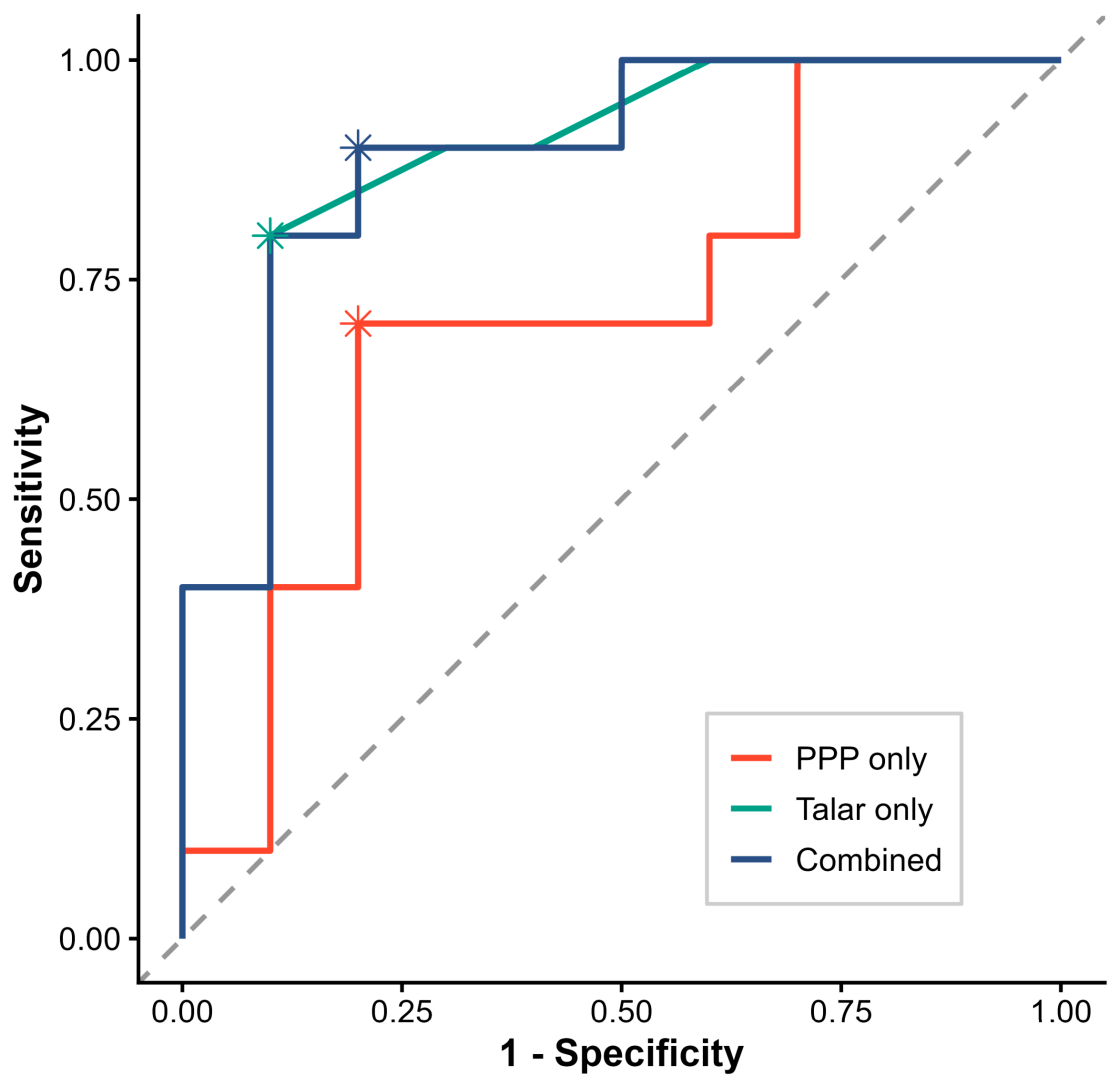
Receiver operating characteristic (ROC) curves for discrimination of delayed remission (time to remission > 132 days) by medial midfoot peak plantar pressure alone, talar declination alone, and the combined model. Talar declination alone and the combined model produced identical area under the curve (AUC) estimates (0.89), and their curves are largely overlapping. Asterisks indicate the Youden index threshold for each model. For the combined model, the threshold maximizing sensitivity was selected (probability threshold 0.464, sensitivity 0.90, specificity 0.80). Delayed remission defined by cohort median split (*n* = 10 per group).

In this exploratory cohort, talar declination alone showed strong discrimination for delayed remission (AUC 0.89, 95% CI 0.74 to 1.00; sensitivity 0.80, specificity 0.90 at threshold 25°; Table 4). Medial midfoot peak plantar pressure alone showed moderate discrimination (AUC 0.71, 95% CI 0.47 to 0.95; Table 4). Combining medial midfoot peak plantar pressure with talar declination produced an identical AUC to talar declination alone (0.89, 95% CI 0.74 to 1.00), confirmed by DeLong testing (*p* = 1.00). The combined model produced an alternative Youden threshold with higher sensitivity (0.90) and lower specificity (0.80). The difference in AUC between talar declination alone and medial midfoot peak plantar pressure alone did not reach statistical significance (*p* = 0.194), though the study was not powered to detect such differences. Results were consistent in sensitivity analyses using lateral midfoot peak plantar pressure and Meary’s angle in place of the primary predictors (Table A2). ROC curves for all three primary models are shown in Figure 2.

### 3.8. Sensitivity Analysis

Given that the cohort included two participants with non-midfoot CNO at diagnosis (one with Sanders–Frykberg pattern I metatarsal/phalangeal, and one with pattern IV ankle/subtalar), sensitivity analyses were performed excluding these participants (*n* = 18) to examine whether findings were sensitive to anatomical heterogeneity within the cohort. Radiographic angle correlations with time to remission were largely unchanged (talar declination ρ = 0.71, Meary’s angle ρ = −0.64). Midfoot peak plantar pressure correlations were marginally stronger in the restricted cohort. Talar declination AUC was slightly lower in the restricted cohort (0.80 vs. 0.89). Full results are presented in Table A3.

To explore whether time to remission varied by anatomical extent of disease, participants were grouped descriptively as isolated midfoot disease (Pattern II or III, *n* = 11), multiple-pattern involvement (*n* = 7), or isolated non-midfoot disease (Pattern I or IV, *n* = 2). Median time to remission was 103 days (range 89–285) for isolated midfoot, 133 days (range 109–260) for multiple-pattern, and 147 and 161 days for the two non-midfoot cases (Mann–Whitney *p* = 0.113). Median talar declination at diagnosis was 24° in both the isolated midfoot and multiple-pattern groups.

## 4. Discussion

No reliable markers currently exist to predict time to remission in active CNO at the time of diagnosis. The present study addressed this gap by examining radiographic deformity and barefoot plantar pressure as candidate prognostic markers. Talar declination at diagnosis was positively associated with time to remission, such that greater deformity was associated with longer time to remission. Talar declination demonstrated good discriminative performance for delayed remission, though this should be interpreted cautiously given the sample size. Peak plantar pressure showed a consistent positive association with time to remission, with higher midfoot peak pressure associated with longer time to remission but did not add prognostic value beyond radiographic assessment alone in our study. These findings suggest that quantified radiographic deformity at diagnosis may provide clinically useful prognostic information, pending validation in larger cohorts.

### 4.1. Plantar Pressure and Remission

Midfoot peak plantar pressure at diagnosis showed a consistent positive association with time to remission but did not reach statistical significance. The active phase, driven by uncontrolled inflammation [35] may introduce variability in plantar pressure values through edema, gait alteration, and evolving structural instability. These factors provide a plausible explanation for the weaker prognostic signal from pressure at diagnosis, though direct evidence for this in CNO is lacking. By contrast, radiographic angles capture structural deformity independently of the acute inflammatory state, which may explain their stronger prognostic signal at this timepoint. Barefoot midfoot peak plantar pressure in established chronic CNO deformity has been reported at 756 kPa, compared with 146 kPa in high-risk neuropathic feet without CNO [18]. This suggests that once deformity consolidates, structural severity may become the primary determinant of the plantar pressure distribution. Elevated midfoot peak pressure has also demonstrated discriminative performance for ulceration risk in established CNO deformity [14]. In the present study, however, peak plantar pressure at CNO diagnosis did not add prognostic value beyond radiographic assessment. Whether this reflects the inherent variability of pressure values during the active inflammatory phase, or a genuinely limited prognostic signal at this timepoint, warrants investigation in larger prospective studies.

### 4.2. Radiographic Deformity and Remission

Greater sagittal plane deformity at diagnosis was associated with longer time to remission, with three radiographic angles reaching significance after correction for multiple comparisons. Talar declination and Meary’s angle were highly collinear, reflecting the same underlying medial column collapse, while the calcaneal-fifth metatarsal angle extended this pattern to the lateral column. Both Meary’s angle and the calcaneal-fifth metatarsal angle have previously been associated with ulceration risk in established CNO midfoot deformity [13,16]. The present findings suggest these same measures of sagittal plane deformity may also carry prognostic relevance for the active episode.

Greater talar declination indicates greater anterior displacement of the talus into the midfoot, consistent with more extensive structural deformity of the midfoot at diagnosis. More severe deformity at diagnosis may take longer to reach the clinical and radiographic stability required for remission. Participants with talar declination at or above the cohort median of 24° had a median time to remission of 150 days compared with 100 days in those below, representing a potential 50-day difference in offloading duration. This estimate is based on a small sample and should be interpreted as illustrative rather than a precise prediction for individual patients. Greater talar declination at diagnosis may also reflect overall disease severity rather than an independent effect on remission time. Eichenholtz stage was available as a severity classification, but the small number of stage 0 cases (*n* = 6) limited any meaningful comparison between stages. Time to remission did not differ clearly by anatomical pattern, and talar declination at diagnosis was similar between the isolated midfoot and multiple-pattern groups, indicating the deformity–remission association was not simply a reflection of anatomical extent of disease, though subgroup sizes were too small to draw firm conclusions.

Meary’s angle demonstrated comparable discriminative performance to talar declination, with the difference in AUC not reaching statistical significance (DeLong *p* = 0.621), consistent with the high collinearity between the two measures (Spearman ρ = −0.94) and their shared dependence on the same underlying sagittal plane deformity. Although talar declination was selected as the primary predictor on the basis of its marginally stronger correlation with time to remission, the sensitivity analysis confirmed that discriminative performance was not meaningfully affected by which measure was used. These findings suggest that quantified sagittal plane deformity at diagnosis, whether measured as talar declination or Meary’s angle, may help identify patients likely to require prolonged offloading.

### 4.3. Clinical Implications

Weight-bearing lateral radiographs are recommended at diagnosis of active CNO [11] and are standard practice in specialist diabetes foot centers. Measurement of talar declination or Meary’s angle from routinely acquired radiographs may therefore represent a low-cost addition to existing clinical assessment. These measurements could be performed by an adequately trained clinician or incorporated into structured radiological reporting, depending on local resources and clinical workflow. If confirmed in larger studies, routine radiographic assessment at diagnosis could support discussions with patients about the likely treatment duration and clinical decision making around offloading duration and monitoring intensity. Median time to remission in this cohort was 132 days, consistent with reported treatment durations of four to six months from US and European centers [36,37], and shorter than the nine to twelve months reported in UK studies [38,39]. Variation in reported treatment durations across the literature has been attributed to differences in remission definitions, monitoring protocols, and offloading approaches [29]. Prolonged immobilization substantially affects mobility, employment, and quality of life, making estimation of likely treatment duration at diagnosis potentially clinically relevant [19,20,40].

### 4.4. Strengths and Limitations

All participants were managed by the same clinical team using consistent protocols throughout the episode of care, with total contact casting as the standard offloading treatment, providing homogeneity across the cohort. Radiographic images were pseudonymized and presented in randomized order prior to measurement, reducing the risk of measurement bias. Inter-rater reliability was assessed by an independent blinded musculoskeletal radiologist, and agreement was good to excellent across all measurements. Radiographic data were available at both diagnosis and remission for all but one participant, enabling assessment of change over the episode. Plantar pressure measurements were obtained in all participants at a median 12 days after diagnosis (IQR 2 to 21 days), consistent with representative measurement of the active inflammatory phase. This also provided regional barefoot pressure data in a cohort where such measurements are rarely reported.

Despite this, although most measurements were obtained within three weeks of diagnosis, a small number were obtained later, up to 59 days, which may have introduced some variability in the plantar pressure values. The sample size of 20 restricts statistical power and confidence intervals were wide across analyses. Findings should therefore be considered exploratory and hypothesis-generating. The retrospective design introduces risk of selection bias and limits control over data collection.

Remission was defined by multidisciplinary consensus rather than a single validated criterion, consistent with current clinical practice and international guidelines [11]. As all participants were managed at a single center by the same clinical team, variability in remission assessment was likely limited. The absence of a validated objective remission criterion nevertheless remains a methodological challenge in CNO research. Time from symptom onset to diagnosis was not available, and it is therefore unclear whether the degree of deformity at diagnosis reflects diagnostic delay, inherent disease severity, or both.

The 132-day cut-off reflects this cohort’s own distribution of remission times rather than an externally validated definition and should not be interpreted as a clinically meaningful threshold for delayed remission. Youden index thresholds for plantar pressure and talar declination were derived from a median split outcome definition and closely approximated the cohort median predictor values, limiting independent clinical interpretability. A clinically applicable threshold for either measure cannot be determined from this sample size and would require prospective validation in a larger cohort. Medial midfoot peak plantar pressure was selected as the primary plantar pressure predictor based on its marginally stronger association with time to remission compared to lateral midfoot peak plantar pressure. A sensitivity analysis confirmed that the combined model result was not significantly affected by midfoot region selection (Table A2).

The majority of participants were enrolled in a registered randomized controlled trial investigating denosumab for active CNO at the same institution. Treatment allocation is unknown to the investigators of the present analysis, but the effect of denosumab on time to remission cannot be excluded. This represents a source of confounding that cannot be quantified.

These findings are exploratory and prospective validation in larger cohorts is needed before talar declination can be considered a clinically applicable prognostic marker. Whether the directional association observed for midfoot peak plantar pressure reaches significance in adequately powered studies also warrants investigation, given that pressure measurement at diagnosis is feasible and could complement radiographic assessment in routine clinical care. Longitudinal studies examining how the relationship between structural deformity and plantar pressure evolves from the active phase through to established deformity would further advance the understanding of the biomechanical consequences of CNO. Earlier offloading before overt radiographic abnormalities have developed has been associated with less structural disruption and shorter treatment duration [8,9]. Plain radiographic measurement may underestimate structural disruption in early-stage disease, and the additional prognostic value of advanced imaging modalities such as magnetic resonance imaging (MRI) or dual-energy CT (DECT) warrants investigation [41,42]. Whether combining diagnostic timing with quantified radiographic severity at presentation could further refine prognostic accuracy, and support more individualized patient counseling, merits prospective investigation.

## 5. Conclusions

In this exploratory cohort, talar declination at diagnosis was associated with time to remission in active CNO and showed a high point estimate for discriminative performance for delayed remission. Plantar pressure showed a consistent directional association but did not reach statistical significance and did not improve discriminative performance when combined with radiographic assessment. Radiographic angle measurement at diagnosis, imaging already part of routine clinical practice, may provide a low cost and accessible means of supporting prognostic discussions with patients and informing decisions around offloading duration. Prospective validation in larger cohorts is needed before these findings can be applied clinically.

## Figures and Tables

**Table 1:** Baseline characteristics of the included participants.

Variable	*N* = 20
**General characteristics**	
Sex, female/male	50% (10)/50% (10)
Age, years	58.2 ± 12.2
BMI, kg/m^2^	29.7 ± 4.8
Smoking status	
Never	47.4% (9)
Current	31.6% (6)
Ex-smoker	21.1% (4)
CNO laterality, left/right	50% (10)/50% (10)
**Diabetes**	
Type 1/Type 2/Other	10% (2)/85% (17)/5% (1)
Diabetes duration, years ^a^	16.5 [6.2, 20.0]
HbA1c, mmol/mol ^a^	61.0 [48.8, 69.0]
LDL, mmol/L	2.7 ± 1.0
**Comorbidities**	
CKD stage	
Stage 1	35% (7)
Stage 2	45% (9)
Stage 3	20% (4)
Retinopathy	35% (7)
Peripheral artery disease ^b^	15% (3)
Peripheral neuropathy	100% (20)
**Foot ulceration history**	
Both limbs	50% (10)
Index limb only	10% (2)
Contralateral limb only	10% (2)
Neither limb	20% (4)
Unknown	10% (2)
**CNO characteristics**	
Eichenholtz stage	
Stage 0	30% (6)
Stage 1	70% (14)
Sanders–Frykberg pattern of involvement	
Pattern I (metatarsal/phalangeal)	5% (1)
Pattern II (tarsometatarsal)	50% (10)
Pattern III (midtarsal)	5% (1)
Pattern IV (ankle/subtalar)	5% (1)
Multiple patterns	35% (7)

Note: ^a^ Presented as median [interquartile range, IQR]. All other continuous variables presented as mean ± SD. ^b^ Peripheral artery disease status unknown in two participants (10%). Missing data: diabetes duration *n* = 2 (10%), BMI *n* = 2 (10%), smoking status *n* = 1 (5%). BMI, body mass index; CNO, Charcot neuro-osteoarthropathy; CKD, chronic kidney disease; HbA1c, glycated hemoglobin; IQR, interquartile range; LDL, low-density lipoprotein; SD, standard deviation.

**Table 2:** Peak barefoot plantar pressure (kPa) at CNO diagnosis and remission. Values at diagnosis are additionally stratified by remission group.

Region	Diagnosis *n* = 20 Median [IQR]	Remission *n* = 18 Median [IQR]	Diagnosis Plantar Pressures by Remission Group	
			Fast (≤132 Days) *n* = 10 Median [IQR]	Delayed (>132 Days) *n* = 10 Median [IQR]
Total foot ^†^	698 [565, 987]	671 [519, 966]	651 [390, 838]	711 [625, 1004]
Toes				
Hallux	323 [122, 617]	476 [210, 728]	247 [96, 532]	406 [160, 804]
2nd toe	182 [110, 349]	180 [144, 306]	182 [123, 326]	189 [101, 411]
3rd–5th toes	137 [36, 326]	102 [39, 277]	117 [50, 158]	172 [37, 373]
Metatarsal heads				
1st metatarsal head	350 [129, 641]	496 [238, 720]	368 [161, 634]	301 [135, 602]
2nd metatarsal head	251 [172, 383]	281 [214, 398]	211 [172, 286]	322 [179, 428]
3rd metatarsal head	254 [188, 361]	252 [231, 316]	234 [179, 261]	272 [215, 406]
4th metatarsal head	191 [165, 287]	204 [196, 317]	191 [152, 263]	192 [171, 317]
5th metatarsal head	161 [96, 277]	187 [89, 400]	137 [87, 256]	198 [132, 303]
Midfoot				
Medial midfoot ^†^	131 [98, 192]	142 [102, 197]	123 [80, 133]	159 [123, 215]
Lateral midfoot ^†^	167 [121, 264]	146 [112, 215]	129 [97, 163]	232 [167, 354]
Hindfoot				
Medial hindfoot	252 [209, 299]	267 [224, 313]	226 [201, 289]	264 [243, 296]
Lateral hindfoot	244 [203, 281]	252 [216, 273]	216 [192, 281]	249 [243, 274]

Values are median [IQR]. ^†^ Pre-specified primary regions for correlation and receiver operating characteristic (ROC) analyses. IQR, interquartile range; kPa, kilopascal.

**Table 3:** Spearman rank correlation coefficients between either peak plantar pressure or radiographic angles at diagnosis and time to remission.

Variable	*n*	Spearman ρ	*p*-Value	FDR-Adjusted *p*
Peak plantar pressure at diagnosis				
Total foot	20	0.173	0.465	0.465
Medial midfoot ^†^	20	0.421	0.064	0.158
Lateral midfoot ^†^	20	0.373	0.105	0.158
Radiographic angles at diagnosis				
Talar declination (°) ^‡^	20	0.759	<0.001	<0.001
Meary’s angle (°) ^‡^	20	−0.686	0.001	0.002
Calcaneal–5th metatarsal angle (°) ^‡^	17	−0.645	0.005	0.008
Calcaneal pitch (°)	20	−0.388	0.091	0.114
Cuboid height (mm)	20	−0.238	0.312	0.312

FDR correction (Benjamini–Hochberg) applied separately to plantar pressure correlations (three comparisons) and radiographic angle correlations (five comparisons). ^†^ Pre-specified primary regions for plantar pressure analysis. ^‡^ Remained statistically significant after FDR correction (FDR-adjusted *p* < 0.05). FDR, false discovery rate.

**Table 4:** Discriminative performance of peak plantar pressure and radiographic angles at CNO diagnosis for delayed remission.

Predictor	*n*	AUC (95% CI)	Threshold	Sensitivity	Specificity	PPV	NPV	LR+	LR−
Peak plantar pressure									
Total foot	20	0.67 (0.41 to 0.92)	607	0.90	0.50	0.64	0.83	1.80	0.20
Medial midfoot	20	0.71 (0.47 to 0.95)	141	0.70	0.80	0.78	0.73	3.50	0.38
Lateral midfoot	20	0.77 (0.55 to 0.99)	173	0.70	0.80	0.78	0.73	3.50	0.38
Radiographic angles									
Talar declination (°)	20	0.89 (0.74 to 1.00)	25°	0.80	0.90	0.89	0.82	8.00	0.22
Meary’s angle (°)	20	0.86 (0.69 to 1.00)	−6°	1.00	0.70	0.77	1.00	3.33	0.00 §
Calcaneal–5th metatarsal angle (°)	17	0.75 (0.51 to 0.99)	23.5°	0.78	0.62	0.67	0.75	2.07	0.36
Calcaneal pitch (°)	20	0.64 (0.37 to 0.91)	19.5°	0.80	0.60	0.67	0.75	2.00	0.33
Cuboid height (mm)	20	0.63 (0.37 to 0.89)	13.5	1.00	0.30	0.59	1.00	1.43	0.00

Thresholds identified by the Youden index. Delayed remission is defined as time to remission exceeding 132 days. § LR− of 0.00 reflects perfect sensitivity at the Youden threshold. Thresholds and associated performance estimates are derived from a small sample (*n* = 20) and are exploratory rather than clinically validated values. AUC, area under the curve; CI, confidence interval; LR+, positive likelihood ratio; LR−, negative likelihood ratio; NPV, negative predictive value; PPV, positive predictive value.

**Table 5:** Paired radiographic angle measurements at diagnosis and remission.

Angle	*n* (Diagnosis)	Diagnosis Mean (SD)	*n* (Remission)	Remission Mean (SD)	*n* (Paired)	Change (Remission − Diagnosis) Mean (SD)
Talar declination (°)	20	25.4 (7.6)	19	26.4 (7.0)	19	1.4 (3.6)
Meary’s angle (°)	20	−9.7 (11.4)	19	−11.0 (11.5)	19	−1.6 (4.1)
Calcaneal–5th metatarsal angle (°)	17	23.5 (7.4)	17	22.4 (7.2)	16	−1.5 (3.4)
Calcaneal pitch (°)	20	18.3 (5.8)	20	17.3 (6.2)	20	−1.0 (2.9)
Cuboid height (mm)	20	9.3 (4.9)	20	8.9 (5.1)	20	−0.5 (1.5)

Values are mean (SD). Change calculated as remission minus diagnosis value. Calcaneal–5th metatarsal angle: *n* = 17 at diagnosis and remission; *n* = 16 paired, as landmarks were unreadable in four participants across the two timepoints (two missing at both timepoints, one at diagnosis only, one at remission only).

## Data Availability

The data presented in this study are not publicly available due to privacy and ethical restrictions. Data may be available from the corresponding author upon reasonable request and with permission of the Medical Ethics Review Committee (MERC) of Amsterdam UMC.

## Abbreviations

The following abbreviations are used in this manuscript:

CNO	Charcot neuro-osteoarthropathy
ROC	Receiver operating characteristic
PPP	Peak plantar pressure
AUC	Area under the curve
CI	Confidence interval
IQR	Interquartile range
SD	Standard deviation
ICC	Intraclass correlation coefficient
FDR	False discovery rate
BMI	Body mass index
HbA1c	Glycated hemoglobin
LDL	Low-density lipoprotein
CKD	Chronic kidney disease
PPV	Positive predictive value
NPV	Negative predictive value
LR+	Positive likelihood ratio
LR-	Negative likelihood ratio
MRI	Magnetic resonance imaging
DECT	Dual-energy CT

## Appendix A

### Appendix A.1. Full Correlation Results

**Table A1:** Spearman rank correlations between peak plantar pressure regions and radiographic angles at diagnosis.

PPP Region	Radiographic Angle	*n*	Spearman ρ	*p*-Value	FDR-Adjusted *p*
Total foot					
	Talar declination (°)	20	0.334	0.150	0.205
	Meary’s angle (°)	20	−0.324	0.164	0.205
	Calcaneal–5th metatarsal angle (°)	17	−0.202	0.437	0.468
	Calcaneal pitch (°)	20	−0.278	0.235	0.271
	Cuboid height (mm)	20	−0.325	0.162	0.205
Medial midfoot ^†^					
	Talar declination (°)	20	0.484	0.030	0.112
	Meary’s angle (°)	20	−0.605	0.005	0.075
	Calcaneal–5th metatarsal angle (°)	17	−0.482	0.050	0.150
	Calcaneal pitch (°)	20	−0.52	0.019	0.095
	Cuboid height (mm)	20	−0.552	0.012	0.090
Lateral midfoot ^†^					
	Talar declination (°)	20	0.347	0.134	0.205
	Meary’s angle (°)	20	−0.391	0.088	0.205
	Calcaneal–5th metatarsal angle (°)	17	−0.388	0.124	0.205
	Calcaneal pitch (°)	20	−0.164	0.488	0.488
	Cuboid height (mm)	20	−0.361	0.118	0.205

FDR correction (Benjamini–Hochberg) applied across all 15 pairwise comparisons. No associations remained significant after FDR correction. All plantar pressure measurements obtained at diagnosis. Calcaneal–5th metatarsal angle available in 17 of 20 participants. ^†^ Pre-specified primary regions for plantar pressure analysis. FDR, false discovery rate; PPP, peak plantar pressure.

### Appendix A.2. Sensitivity Analysis Using Alternative Predictor Measures

**Table A2:** Sensitivity analyses: discriminative performance for delayed remission using (1) lateral midfoot plantar pressure in place of medial midfoot plantar pressure, and (2) Meary’s angle in place of talar declination.

Model	AUC (95% CI)	DeLong *p* vs. Talar Alone
Lateral midfoot PPP sensitivity analysis		
Lateral midfoot PPP only	0.77 (0.55 to 0.99)	0.379
Talar declination only	0.89 (0.74 to 1.00)	—
Combined (lateral midfoot PPP + talar)	0.95 (0.87 to 1.00)	0.262
Meary’s angle sensitivity analysis		
Meary’s angle only	0.86 (0.69 to 1.00)	—
Combined (Meary’s angle + medial midfoot PPP)	0.86 (0.69 to 1.00)	1.00

Delayed remission is defined as time to remission exceeding 132 days. DeLong *p*-values compare the combined model against the radiographic measure alone within each analysis. AUC, area under the curve; CI, confidence interval; PPP, peak plantar pressure.

Two sensitivity analyses were performed. Replacing medial midfoot plantar pressure with lateral midfoot plantar pressure in the combined model produced an AUC of 0.95 (95% CI 0.87 to 1.00), numerically higher than talar declination alone but not significantly different on DeLong testing (*p* = 0.262). Replacing talar declination with Meary’s angle in the combined model produced an AUC of 0.86 (95% CI 0.69 to 1.00), identical to Meary’s angle alone (DeLong *p* = 1.00). The primary model result was consistent across both sensitivity analyses.

### Appendix A.3. Sensitivity Analysis Excluding Non-Midfoot Participants

**Table A3:** Sensitivity analysis excluding participants with non-midfoot CNO (*n* = 18): comparison with primary analysis (*n* = 20).

Variable	Primary Analysis (*n* = 20)	Sensitivity Analysis (*n* = 18)
Peak plantar pressure—Spearman ρ		
Medial midfoot	0.421	0.450
Lateral midfoot	0.373	0.471
Radiographic angles—Spearman ρ		
Talar declination (°)	0.759	0.709
Meary’s angle (°)	−0.686	−0.638
Discriminative performance—AUC		
Medial midfoot plantar pressure	0.71	0.75
Talar declination	0.89	0.88

Sensitivity analysis excluded two participants with non-midfoot CNO at diagnosis (Sanders–Frykberg pattern I and pattern IV). Delayed remission is defined as time to remission exceeding 132 days. AUC, area under the curve.
